# Effects of nanoemulsion of banana (*Musa acuminata*) leaf extract on ALP, osteocalcin, VEGF, and CD31 expression in orthodontic mechanical force application in Wistar rats (in vivo)

**DOI:** 10.1016/j.jtumed.2026.09.015

**Published:** 2026-11-02

**Authors:** Ari Triwardhani, Nurul A.R. Putranti, Rizki Y. Rahmah, Nabila Z. Sannidhya, Ijma G. Putri, I Gusti Aju Wahju Ardani, Ervina R. Winoto, Desy Novianti, Labieb F. Zaky, Ananda F. Putranto, Adya Pramusita, Tengku N.E.b.T.A. Noor, Wibi Riawan, Alexander P. Nugraha

**Affiliations:** a Orthodontic Department, Faculty of Dental Medicine, Universitas Airlangga, Surabaya, East Java, Indonesia; b Faculty of Dental Surgery, Royal College of Surgeon, Edinburgh University, United Kingdom; c Malaysian Armed Forces Dental Officer, 609 Armed Forces Dental Clinic, Kuching, Serawak, Malaysia; d Department of Biomolecular Biochemistry, Faculty of Medicine, Brawijaya University, Malang, East Java, Indonesia; e Division of Globalization Initiative, Liaison Center for Innovative Dentistry, Graduate School of Dentistry, Tohoku University, Sendai, Japan

**Keywords:** Alkaline phosphatase (ALP), Banana leaves (*Musa acuminata* L.), Orthodontic tooth movement, Osteocalcin, Platelet Endothelial Cell Adhesion Molecule-1 (CD31), Vascular endothelial growth factor (VEGF), أوراق الموز (.Musa acuminata L), حركة الأسنان التقويمية, إنزيم الفوسفاتاز القلوي (ALP), الأوستيوكالسين, عامل نمو بطانة الأوعية الدموية (VEGF), جزيء التصاق الخلايا البطانية والصفائح الدموية-1 (CD31)

## Abstract

**Objectives:**

Orthodontic tooth movement (OTM) is driven by mechanical stimuli and regulated by alveolar bone remodeling and the periodontal ligament (PDL). Compressive forces induce angiogenesis in the periodontium by activating vascular endothelial growth factor (VEGF). Alkaline phosphatase (ALP) serves as an early marker of osteoblastic activity and osteocalcin indicates mature bone formation and mineralization. Banana (*Musa acuminata* L.) leaves contain bioactive compounds, including flavonoids, tannins, alkaloids, terpenoids, saponins, and catechins, which possess anti-inflammatory and wound-healing properties that may enhance tissue remodeling. The objective of this study was to analyze the effects of a banana leaf extract nanoemulsion on ALP, osteocalcin, VEGF, and CD31 expression during OTM in Wistar rats (*Rattus norvegicus*).

**Methods:**

Sixty male Wistar rats (aged 16–20 weeks, weighing 250–300 g) were divided into four groups (n = 15/group): K0 (no OTM), K− (OTM only), KP (OTM+banana leaf nanoemulsion), and K+ (OTM+etoricoxib), which were subdivided by observation period on days 14, 21, and 28 (n = 5/subgroup). OTM was induced using a 6 mm nickel–titanium closed coil spring. The banana leaf extract nanoemulsion was administered locally once daily into the gingival sulcus of the first molars using a microliter syringe at a dose of 0.1 mL. Periodontal tissue samples were evaluated using immunohistochemical staining to measure the expression of ALP, osteocalcin, VEGF, and CD31.

**Results:**

The banana leaf nanoemulsion (KP) significantly increased the expression levels of ALP, osteocalcin, VEGF, and CD31 compared with the OTM-only group (K−) after all observation periods. These increased expression levels were associated with enhanced angiogenesis and accelerated osteoblast activity, and bone mineralization in the remodeled periodontium.

**Conclusions:**

Banana leaf extract nanoemulsion effectively accelerated periodontal tissue remodeling by promoting angiogenesis and osteoblast activity during OTM, demonstrating strong potential for use as a supportive therapeutic agent.

## الملخص

**أهداف البحث:**

تحدث حركة الأسنان التقويمية بفعل المحفزات الميكانيكية، وتُنظَّم من خلال إعادة تشكيل العظم السنخي والرباط حول السني. وتؤدي قوى الضغط إلى تحفيز تكوّن الأوعية الدموية في أنسجة دواعم السن من خلال تنشيط عامل نمو بطانة الأوعية الدموية (VEGF). ويُعد الفوسفاتاز القلوي (ALP) مؤشرًا مبكرًا لنشاط بانيات العظم، في حين يُعد الأوستيوكالسين مؤشرًا على نضج تكوّن العظم وتمعدنه. وتحتوي أوراق الموز (.Musa acuminata L) على مركبات حيوية فعالة، تشمل الفلافونويدات، والعفص، والقلويدات، والتربينويدات، والصابونينات، والكاتيكينات، وتمتلك هذه المركبات خصائص مضادة للالتهاب ومحفزة لالتئام الجروح، مما قد يسهم في تعزيز إعادة تشكيل الأنسجة. وهدفت هذه الدراسة إلى تحليل تأثير مستحلب نانوي من مستخلص أوراق الموز في التعبير عن الفوسفاتاز القلوي، والأوستيوكالسين، وعامل نمو بطانة الأوعية الدموية، وCD31 أثناء حركة الأسنان التقويمية في جرذان ويستار (Rattus norvegicus).

**طرق البحث:**

شملت الدراسة 60 جرذًا ذكرًا من سلالة ويستار، تراوحت أعمارها بين 16 و20 أسبوعًا، وأوزانها بين 250 و300 غرام، وقُسِّمت إلى أربع مجموعات، بواقع 15 جرذًا لكل مجموعة: المجموعة K0 دون حركة أسنان تقويمية، والمجموعة −K التي خضعت لحركة الأسنان التقويمية فقط، والمجموعة KP التي خضعت لحركة الأسنان التقويمية مع استخدام المستحلب النانوي لمستخلص أوراق الموز، والمجموعة +K التي خضعت لحركة الأسنان التقويمية مع إعطاء إيتوريكوكسيب. كما قُسِّمت كل مجموعة بحسب مدة المتابعة إلى الأيام 14 و21 و28، بواقع خمسة جرذان في كل مجموعة فرعية. واستُحثت حركة الأسنان التقويمية باستخدام نابض لولبي مغلق من النيكل والتيتانيوم بطول 6 مم. وأُعطي المستحلب النانوي لمستخلص أوراق الموز موضعيًا مرة واحدة يوميًا داخل الميزاب اللثوي للأضراس الأولى باستخدام محقنة ميكرولترية بجرعة 0.1 مل. وتم تقييم عينات أنسجة دواعم السن باستخدام الصبغ المناعي الكيميائي النسيجي لقياس التعبير عن الفوسفاتاز القلوي، والأوستيوكالسين، وعامل نمو بطانة الأوعية الدموية، وCD31.

**النتائج:**

أدى استخدام المستحلب النانوي لمستخلص أوراق الموز في المجموعة KP إلى زيادة ذات دلالة إحصائية في مستويات التعبير عن الفوسفاتاز القلوي، والأوستيوكالسين، وعامل نمو بطانة الأوعية الدموية، وCD31 مقارنةً بالمجموعة التي خضعت لحركة الأسنان التقويمية فقط (−K) خلال جميع فترات المتابعة. وارتبط هذا الارتفاع بتعزيز تكوّن الأوعية الدموية، وتسريع نشاط بانيات العظم، وزيادة تمعدن العظم في أنسجة دواعم السن الخاضعة لإعادة التشكيل.

**الاستنتاجات:**

أظهر المستحلب النانوي لمستخلص أوراق الموز فعالية في تسريع إعادة تشكيل أنسجة دواعم السن من خلال تعزيز تكوّن الأوعية الدموية ونشاط بانيات العظم أثناء حركة الأسنان التقويمية، مما يشير إلى إمكانية استخدامه كعامل علاجي داعم.

## Introduction

Malocclusion is a deviation from normal dental and skeletal development where the alignment of the teeth and jaws fails to achieve functional and aesthetic harmony.^1^ This condition affects the facial appearance as well as influencing oral functions, such as chewing, speaking, and jaw movement, and the stability of the temporomandibular joint.^2^ About one-third of the population exhibit normal occlusion, whereas the remainder exhibit malocclusion to various degrees.^3^ Malocclusion can also cause psychosocial consequences because of altered facial aesthetics, contributing to reduced self-confidence and social discrimination.^4^ (see Figure 1, Figure 2, Figure 3, Figure 4, Figure 5)

**Figure 1:**
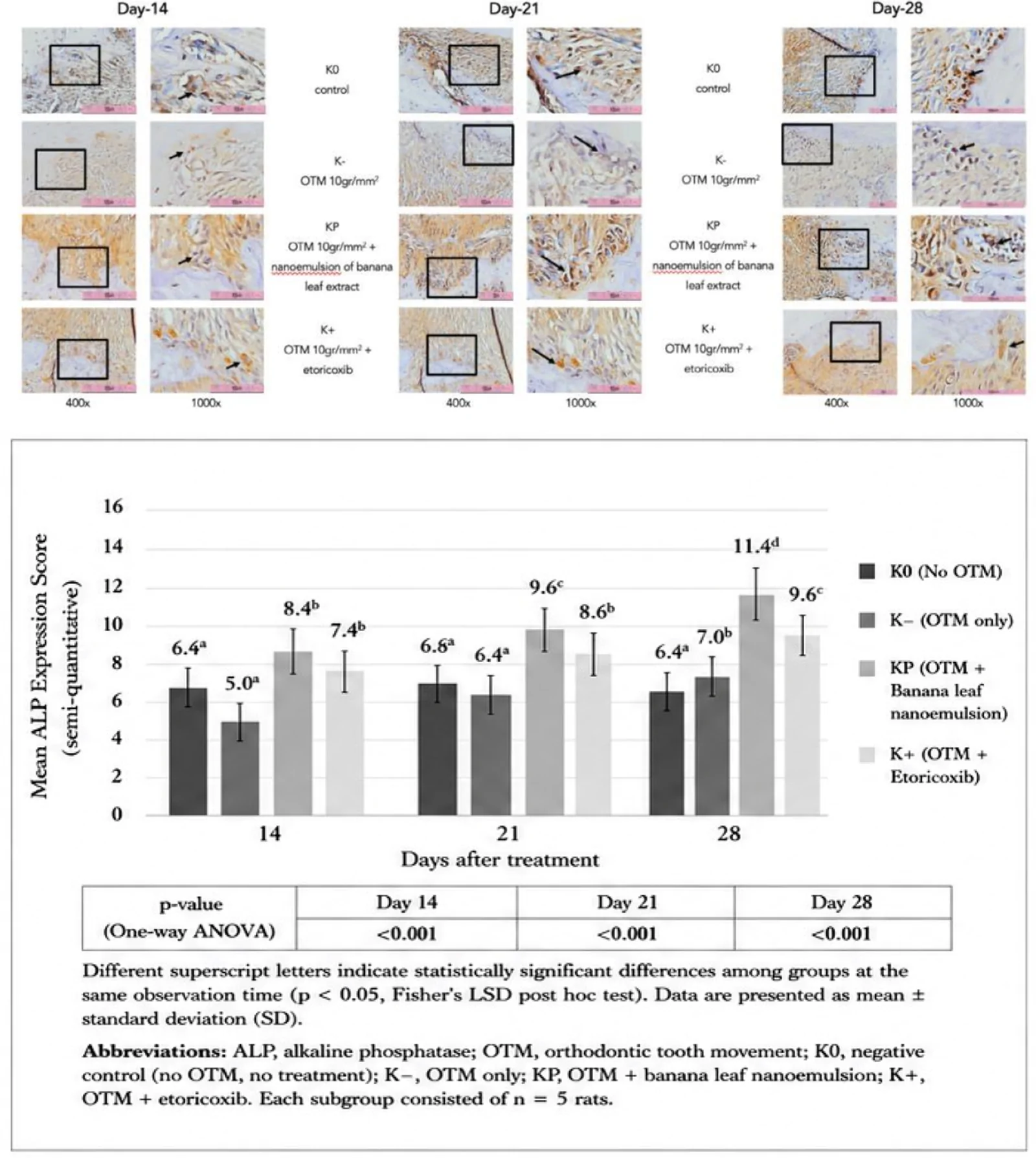
ALP expression. (A) Positive ALP expression shown by the brown color in osteoblasts on the tension side in alveolar bone during orthodontic tooth movement at 400 × magnification and 1000 × under a light microscope. Scale bar = 100 μm. (B) Mean ALP expression (and standard deviation) under each treatment.

**Figure 2:**
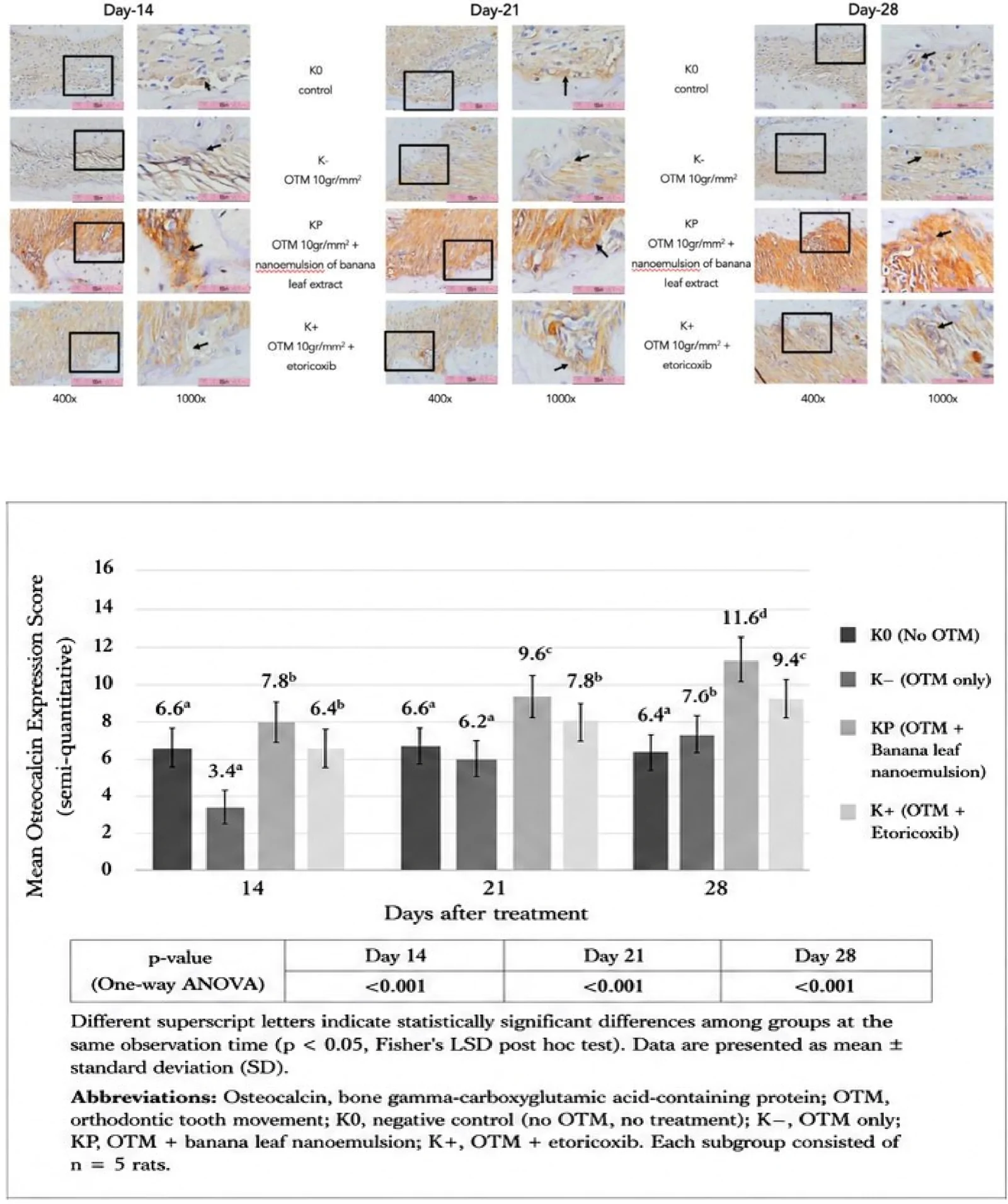
Osteocalcin expression. (A) Positive osteocalcin expression shown by the brown color (black square and arrow) in osteoblasts on the tension side in alveolar bone during orthodontic tooth movement at magnifications of 400 × and 1000 × under a light microscope. Scale bar = 100 μm. (B) Mean osteocalcin expression levels (and standard deviation) under each treatment.

**Figure 3:**
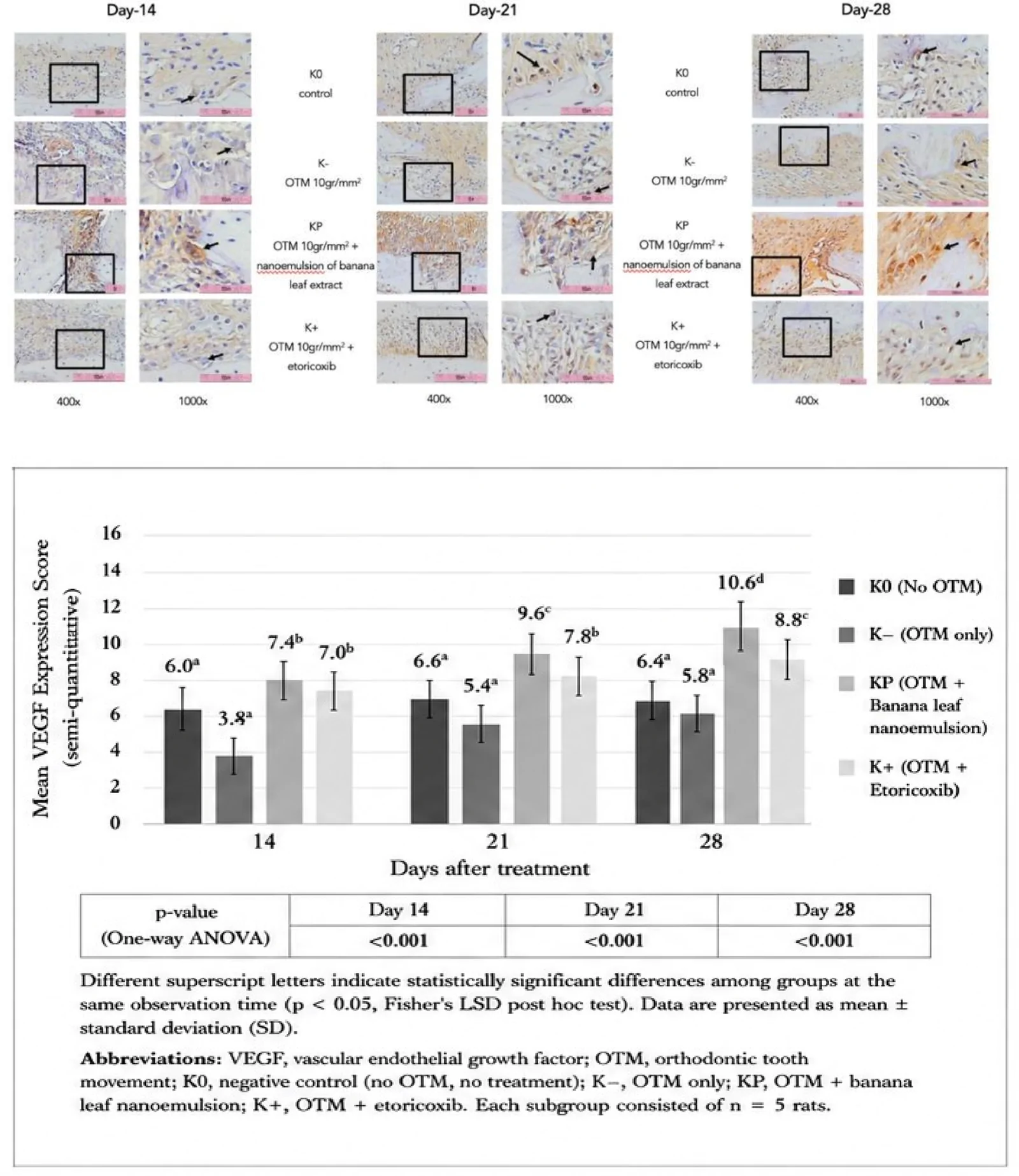
VEGF expression. (A) Positive VEGF expression shown by the brown color (black square and arrow) in osteoblasts on the tension side in alveolar bone during orthodontic tooth movement at magnifications of 400 × and 1000 × under a light microscope. Scale bar = 100 μm. (B) Mean VEGF expression levels (and standard deviation) under each treatment.

**Figure 4:**
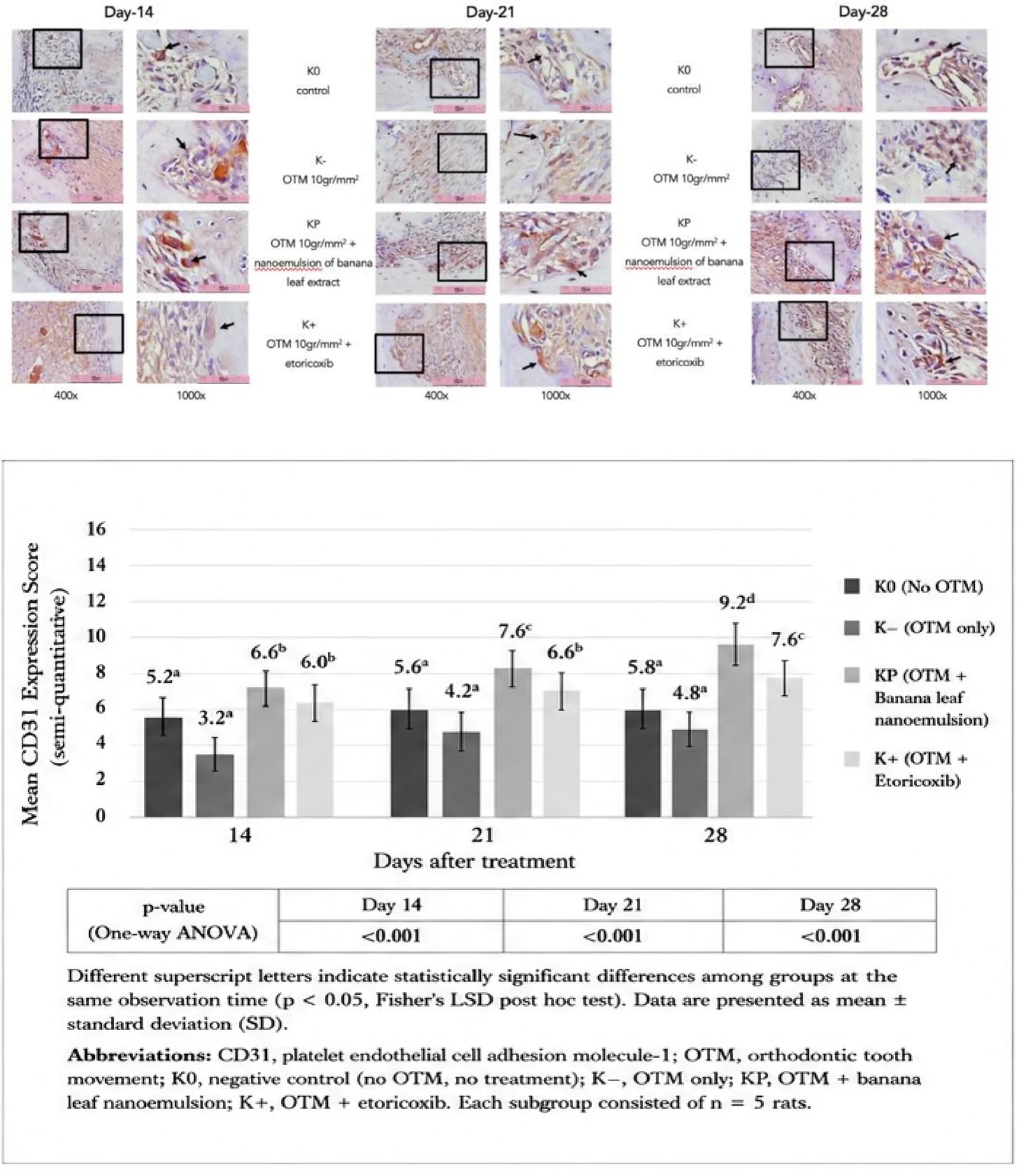
CD31 expression. (A) Positive CD31 expression shown by the brown color (black square and arrow) in endothelial cells on the tension side in alveolar bone under orthodontic tooth movement at magnifications of 400 × and 1000× under a light microscope. Scale bar = 100 μm. (B) Mean CD31 expression levels (and standard deviation) under each treatment.

**Figure 5:**
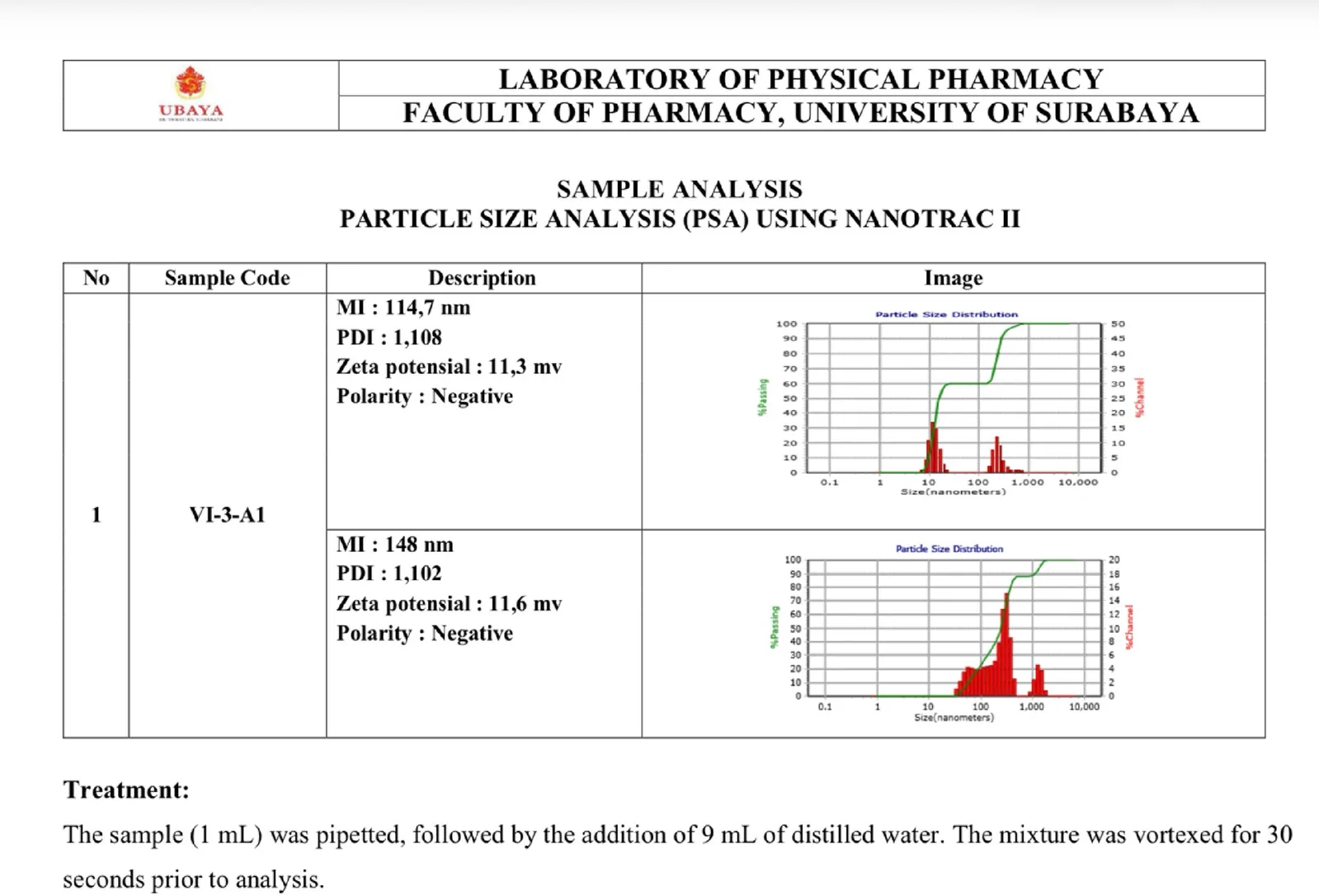
Dynamic light scattering protocol.

In Indonesia, dental and oral health issues are still widespread.^5^ According to National Basic Health Research, the prevalence of dental and oral health issues is 57%, and malocclusion affects 80% of the population.^6^ Malocclusion is the third most prevalent dental issue, following dental caries and periodontal disease.^7^ This high prevalence demonstrates the importance of achieving a better understanding of orthodontic biology in order to develop more efficient and biologically compatible treatment approaches.^8^

The treatment of orthodontic issues can correct tooth alignment and functioning, but also enhance facial aesthetics and improve quality of life.^1^ Orthodontic tooth movement (OTM) depends on the response of the periodontal ligament (PDL) and alveolar bone to the application of external forces. The application of forces in OTM can affect the physiological balance of the dentofacial complex,^9^ leading to cellular responses by osteoclasts, osteoblasts, and cells in the PDL, which simultaneously modify the surrounding tissues to enable controlled tooth movement.^10^

Tooth displacement is an immediate outcome of bone remodeling, which is a coordinated process where bone resorption occurs on the pressure side and apposition of bone on the tension side. On the pressure side, the PDL tightens, causing hypoxia, compression of vascular tissues, and osteoclast precursor recruitment, initiating the resorption of alveolar bone.^1^ On the tension side, PDL stretching stimulates fibroblasts and osteoblast progenitors to produce fresh collagen fibers and alveolar bone.^8^ These changes are reflected in the gingival crevicular fluid where shifts occur in cytokine concentrations, growth factors, neurotransmitters, and arachidonic acid metabolite functions as indicators of ongoing biological activity.^11^

Burstone's OTM model specifies that OTM has three main phases. The initial phase occurs directly following the application of force and it is characterized by rapid tooth displacement due to PDL compression and fluid redistribution. This phase is followed by the lag phase, where little or no visible movement occurs due to the formation of hyalinized tissue and temporary cessation of bone resorption. Tooth movement resumes in the post-lag phase when necrotic tissue is removed and osteoclasts and osteoblasts regain their full activity, enabling progressive and continuous remodeling.^12^

Various biochemical mediators orchestrate this process.^9^ Osteoclastogenesis is promoted by the interaction between receptor activator of nuclear factor-κB ligand (RANKL) and its receptor, RANK.^13^ RANKL is present on osteoblasts, osteocytes, PDL cells, and inflammatory immune cells.^14^ Inflammatory cytokines including IL-1, TNF-α, and prostaglandin E_2_ (PGE_2_) further strengthen osteoclast differentiation and activation.^15^ Controlled osteoclast activity is crucial for facilitating tooth movement but excessive activation can cause adverse effects such as orthodontically induced external root resorption.^16^

Remodeling also depends on angiogenesis because an adequate vascular supply is important for oxygen and nutrient transport, and the removal of metabolic byproducts.^17^ Vascular endothelial growth factor (VEGF) is crucial for the formation of blood vessels and PDL remodeling.^18^ Orthodontic forces cause changes in vascular permeability, neuropeptide release, and endothelial responses, which all play roles in the initial phases of OTM.^19^

Intrinsic biomarkers are valuable in orthodontics for identifying tissue responses and measuring treatment outcomes.^20^ In particular, alkaline phosphatase (ALP) is used as a marker of initial osteoblastic activity, where its expression is frequently enhanced in gingival crevicular fluid during active tooth movement.^21^ Osteocalcin is a non-collagenous protein produced by mature osteoblasts and it is used as an indicator that is highly specific to bone formation and mineralization in the subsequent phases of osteoblastic differentiation.^22^

In recent years, interest has increased in exploring natural bioactive compounds to improve or modulate bone remodeling.^23^ Banana (*Musa acuminata* L.) leaf is a botanical with significant potential pharmacological applications.^24^ Banana is an accessible and sustainable plant resource, and banana leaves contain many active compounds, such as flavonoids, tannins, alkaloids, terpenoids, saponins, and catechins.^25^ These phytochemicals have anti-inflammatory, antioxidant, antimicrobial, and wound-curing properties.^26^ In particular, the phenolic constituents in banana leaves have been shown to aid osteoblast proliferation and differentiation, with potentially advantageous uses in the bone remodeling process.^27^ However, despite these promising biological activities, the use of banana leaf extract in the context of OTM has not been unexplored, and this new field of study has potential clinical relevance.^28^

Etoricoxib is a selective cyclooxygenase-2 (COX-2) inhibitor and it was used in the present study as a reference pharmacological agent because of its well-established anti-inflammatory effects and correlation with the modulation of bone remodeling in OTM.^29^ COX-2 inhibitors affect prostaglandin synthesis, especially PGE_2_, which is a major mediator involved in the initial inflammatory responses, osteoclastogenesis, and PDL remodeling under orthodontic force.^30^ Thus, etoricoxib was employed as a validated pharmacological benchmark for comparison with the biological activity of nanoformulated banana leaf extract.^31^

In contrast to non-selective non-steroidal anti-infla-mmatory drugs, etoricoxib targets the COX-2 isoenzyme without influencing COX-1, thereby reducing adverse gastrointestinal effects and disruption of physiological prostaglandin pathways.^32^ Studies have shown that selective COX-2 inhibition can reduce inflammation and osteoclast formation in the PDL compression side, allowing the OTM magnitude and rate to be modulated.^33^ Due to these properties, etoricoxib was selected as an effective positive control for measuring how an anti-inflammatory agent with known COX-2 specificity affected bone remodeling biomarkers, including ALP, osteocalcin, VEGF, and CD31 expression.^34^

In the present study, etoricoxib was provided orally at a dose of 6 mg/kg body weight in liquid preparation because this dose has been used in preclinical models to effectively inhibit COX-2 without systemic toxicity.^35^ Using etoricoxib as a reference medicine facilitated evaluation of whether banana leaf extract nanoemulsion yielded comparable, lesser, or different modulatory effects on orthodontically induced inflammation, angiogenesis, and bone remodeling.^36^ This comparison was important for validating the biological relevance and potential therapeutic value of the experimental extract.^37^

## Materials and Methods

### Sample preparation

All treatments of experimental animals were conducted according to international ethical guidelines for the treatment and use of laboratory animal. Ethical approval was obtained from the Research Ethics Committee of the Faculty of Dentistry, Universitas Airlangga. All efforts were made to minimize animal suffering and the number of animals used.

### Establishment of rat OTM model

Sixty healthy male Wistar rats (*Rattus norvegicus*), aged 16–20 weeks and weighing 250–300 g, were utilized in this study. Animals were acclimatized for 1 week under controlled environmental conditions (temperature: 22 °C ± 2 °C; humidity: 50–60%; 12 h/12 h light/dark cycle) with ad libitum access to standard laboratory chow and water. Rats were randomly allocated to K0 (no OTM), K− (OTM only), KP (OTM+banana leaf nanoemulsion), and K+ (OTM+etoricoxib) treatments, and further subdivided into 12 experimental subgroups according to the treatment and sacrifice time point (days 14, 21, and 28).

Orthodontic mechanical force (OMF) was applied to induce mesial movement of the maxillary first molars. A 6-mm nickel–titanium (NiTi) closed-coil spring (American Orthodontics Corp., Sheboygan, WI, USA) was anchored between the maxillary incisors and first molars. The device delivered a constant 10 g force, which was confirmed using a tension gauge (American Orthodontics, Corp.). The appliance was secured with 0.07-inch stainless steel ligature wires to ensure stability throughout the experimental period. Animals were examined daily to ensure the integrity of the appliance.

Mature banana (*Musa acuminata*) leaves were collected, washed to remove any debris, air dried, and subsequently oven dried at a controlled temperature until constant weight. The dried leaves were ground into coarse powder and weighed using an analytical balance. Extraction was performed via ethanol maceration. The leaf powder was immersed in 96% ethanol at a solvent-to-powder ratio of 10:1 (v/w) in airtight glass containers and allowed to macerate for 72 h with intermittent agitation to enhance solvent penetration and phytochemical extraction. The filtrate was obtained by sequentially passing the mixture through: (1) coarse mesh, (2) standard filter paper, and (3) Whatman No. 41 filter paper, to yield a clear solution. The filtrate was concentrated utilizing a rotary evaporator at 50 °C, 200 mbar, and 40 g to obtain a viscous crude extract, which was stored at 4 °C for nanoformulation preparation.

The concentrated extract was nanoformulated to enhance its physicochemical stability and bioavailability. Several test extracts were dispersed in an aqueous phase containing appropriate surfactants. The mixture was subjected to high power, high shear combination repeated cycles until a uniform nanoscale emulsion was obtained. Parameters such as the homogenization time, surfactant concentration, and sonication intensity were optimized to obtain stable nano-sized droplets. The nanoemulsion was stored in light-protected vials at a controlled temperature before administration (see Table 1).

**Table 1:** Physicochemical characteristics of banana leaf extract nanoemulsion.

Parameter	Result	Interpretation
Mean particle size (nm)	114.7–118.0	Within the nanoscale range
Polydispersity index	1.102–1.108	Broad particle size distribution
Zeta potential (mV)	–11.3 to –11.6	Negative surface charge
Polarity	Negative	Consistent with zeta potential measurement

The particle size, polydispersity index, and zeta potential of the banana leaf extract nanoemulsion were characterized using a NanoTrac II analyzer (Microtrac, Montgomeryville, PA, USA) based on dynamic light scattering. Before analysis, 1 mL of the nanoemulsion was diluted with 9 mL of distilled water and vortexed for 30 s to ensure adequate dispersion. Measurements were performed in triplicate at room temperature. The mean particle sizes were 114.7 nm–118 nm, the polydispersity index values were 1.108 and 1.102, and the zeta potential values were −11.3 mV and −11.6 mV The measured polydispersity index >1 indicated a highly polydisperse system, suggesting possible aggregation or instability of the nanoemulsion.

Treatment was initiated immediately after orthodontic appliance placement. The banana leaf extract nanoemulsion was administered locally into the gingival sulcus adjacent to the maxillary first molar using a microliter syringe at a dose of 0.1 mL once daily. This local application was maintained until the predetermined sacrifice periods elapsed on days 14, 21, and 28. The reference group received etoricoxib orally at a dose of 6 mg/kg body weight once daily throughout the experimental period.

### Tissue preparation and histological analysis

After sacrificing the animals, the maxillary tissues were separated carefully. Specimens were fixed in 10% neutral-buffered formalin (OneMed, Surabaya, Indonesia) for 4 days. The tissues were then decalcified in 10% EDTA solution for around 1 month, where the solution was changed regularly. The samples were then dehydrated, cleared, and paraffin embedded according to standard histological protocols.

Serial paraffin sections were removed and rehydrated through a graded ethanol series. Antigen retrieval was performed utilizing 0.01 M citrate buffer (pH 6.0) in a microwave with full power (750 W) for around 15 min. Endogenous peroxidase was blocked by using 3% hydrogen peroxide in methanol.

Immunohistochemical staining was conducted to detect ALP, osteocalcin, VEGF, and CD31 using EnVision™+ Dual Link System-HRP detection kits (Dako). The sections were incubated with the primary antibodies under the optimal conditions for each marker. Results were visualized utilizing 3,3′-diaminobenzidine as the substrate, before counterstaining the slides with hematoxylin. Positive and internal controls were included in every batch for staining.

Two independent microscopists who were blinded to the identities of the samples assessed immunostaining under a light microscope at 200 × magnification. Cytoplasmic staining was assessed to determine ALP, osteocalcin, VEGF, and CD31 expression levels. The intensity of staining was measured according to chromogen deposition, where darker colors indicated higher protein expression levels. Any differences of opinion were resolved by consensus.

Three serial sections were analyzed from each animal. Five non-overlapping microscopic fields from the tension side adjacent to the PDL were examined under 400 × magnification. Positive immunostaining was identified as brown cytoplasmic staining. Quantification was performed using a semi-quantitative scoring system based on the number of positively stained cells. Two blinded independent observers evaluated all specimens, and interobserver reliability was assessed using Cohen's kappa coefficient.

### Statistical analysis

Statistical analyses were performed using IBM SPSS Statistics software (version X.X; IBM Corp., Armonk, NY, USA). Results were expressed as the mean ± standard deviation. Normality was tested and confirmed by using the Shapiro–Wilk test. Differences in results were determined using one-way analysis of variance (ANOVA). After detecting a statistically significant difference (*p* < 0.001), Fisher's least significant difference (LSD) post hoc test was performed for pairwise comparisons between groups.

## Results

The nanoemulsion was characterized before application in biological evaluations. The mean particle sizes of 114.7 nm and 148 nm confirmed successful formulation within the nanoscale range. The corresponding polydispersity index values of 1.108 and 1.102 indicated a relatively broad particle size distribution. The zeta potential values of −11.3 mV and −11.6 mV demonstrated the negative polarity of the particles. These findings confirmed the successful formation of a nano-sized banana leaf extract dispersion system, which was subsequently used for in vivo evaluation of osteogenic and angiogenic marker expression during OTM.

Statistical analyses demonstrated that there were significant differences in the expression levels of the indicators among the experimental groups after the three test periods (14, 21, and 28 days). The ALP and osteocalcin expression levels increased most under KP. The ALP values in this group increased greatly after 14, 21, and 28 days (8.4 → 9.6 → 11.4) compared with those under K+. The pattern was similar for osteocalcin, with the highest expression levels under KP after 14, 21, and 28 days (7.8 → 9.6 → 11.6). By contrast, the expression levels were lowest and remained stable in the untreated control (K0), and only small increases were found under K−. These findings suggest that the banana leaf extract support the osteogenic activity better than OMF alone and it was more effective than the reference medicine etoricoxib.

The Shapiro–Wilk test results showed that all ALP and osteocalcin data in the 12 groups were normally distributed (*p* > 0.05). The variance of homogeneity assumption was also satisfied for both biomarkers according to Levene's test (ALP: *p* = 0.995; and osteocalcin: *p* = 0.898). Thus, one-way ANOVA was suitable for comparative analysis among the treatment groups.

The average ALP expression levels differed significantly among treatments (*p* < 0.001). The ALP expression levels were consistently low under K0 at all time points, and the increases were only moderate under K−. The ALP expression levels were highest under KP at every time point, with the greatest levels on day 28 (average = 11.40). The ALP expression levels were higher under K+ compared with K− and K0, but the levels were still lower than those under KP. These results show that the banana leaf extract significantly increased ALP expression during OTM, suggesting that it promoted osteoblastic activity.

The osteocalcin expression levels also differed significantly among treatments (*p* < 0.001). The highest average osteocalcin expression levels after all periods were found under KP (highest level of 11.60 on day 28). Moderate increases were found under K−, and the levels remained stable or decreased under K0. The osteocalcin expression levels were higher under K+ compared with K0 and K−, but they were still lower than those under KP. These results indicate that the banana leaf extract enhanced osteoblastic maturation and bone matrix mineralization more efficiently than etoricoxib.

Post hoc comparisons of the differences among treatments showed that the differences were significant among the ALP and osteocalcin expression levels under KP and those under K0 and K− (*p* < 0.001). On day 28, the levels under KP were significantly different compared with those under K0 and K−. The differences under KP and K+ were not significant on day 21 (*p* = 0.258) and the significance was borderline on day 28 (*p* = 0.045), indicating the comparable efficacy of the banana leaf extract and etoricoxib in stimulating ALP expression. The osteocalcin expression levels were significantly higher under KP than those under K0 and K− on days 21 and 28 (*p* < 0.001). On day 28, the osteocalcin expression levels under KP were significantly different from those under K+ (*p* = 0.012) *p* < 0.001

The banana leaf extract clearly obtained the highest ALP and osteocalcin expression levels at all time points. The expression levels of osteogenic markers increased significantly from day 14–28, with the greatest effects under KP. The effects of etoricoxib were evident but it was less effective than the extract, particularly on the long-term expression of osteocalcin. The significant differences among treatments demonstrated that the banana leaf extract increased bone remodeling during OTM.

Descriptive statistics were calculated to compare the mean expression levels of VEGF and CD31, simplifying the analysis of the heightened expression patterns of these two markers after banana leaf extract injection during OTM. The expression levels of VEGF and CD31 were stable from day 14–28 under K0, whereas sharp increases occurred before returning to lower levels under K− (3.8 → 5.4 → 5.8). VEGF expression increased most under KP during OTM (7.4 → 8.6 → 10.6), indicating its effectiveness. The VEGF expression levels increased under K+ (7 → 7.8 → 8.8), but they were still lower than those under KP. CD31 expression increased gradually under K− (4.2 → 5.4 → 7.6), indicating that the orthodontic force stimulated vascularization. The greatest increases in the CD31 expression levels occurred under KP (9 → 10 → 11), demonstrating high CD31 expression during tooth movement. The CD31 expression levels also increased under K+ (6 → 7.8 → 8.6), but the levels were higher under KP.

The Shapiro–Wilk test of normality results showed that VEGF and CD31 expression levels were normally distributed under all treatments at different observation times (*p* > 0.05.) The Levene's test results to assess homogeneity of variance showed that the *p*-values were 0.427 (>0.05) for the VEGF expression levels and 0.950 (>0.05) for the CD31 expression levels, demonstrating the homogeneity of the variances under different treatments. Thus, the assumptions of normality and homogeneity were satisfied for the data, so one-way ANOVA was conducted to compare the differences in the VEGF and CD31 expression levels.

The one-way ANOVA test results showed that the VEGF and CD31 expression levels differed highly significantly between treatments (*p* < 0.001). The increases in the VEGF and CD31 expression levels were highest under KP, and the ANOVA results supported the hypothesis that the banana leaf extract could enhance the expression of VEGF and CD31 in the tension site during OTM.

Following ANOVA, post hoc LSD tests were conducted to confirm the differences in VEGF and CD31 expression between groups. The post hoc LSD test results showed that the VEGF expression levels differed greatly under the treatment groups, particularly under KP and K−. The VEGF expression levels under K− and KP differed significantly from those under the other treatments at all observation times. The expression levels under KP on day 28 differed most from those under the other treatments (*p* < 0.001), demonstrating that the time with the highest elevated growth and VEGF expression under KP occurred on day 28. Thus, the significant differences detected by LSD tests confirmed that the banana leaf extract promoted much stronger increases in VEGF expression compared with the other treatments.

The post hoc LSD tests showed that the CD31 expression levels under KP differed significantly compared with those under the other treatments, especially on days 21 and 28, indicating that the banana leaf extract was most effective at increasing CD31 expression. One-way ANOVA detected statistically significant differences in CD31 expression between treatments at all observation times (*p* < 0.001). Therefore, Fisher's LSD post hoc test was used to conduct multiple comparisons.

## Discussion

OTM is a physiological response of the facial bones to an external stimulus that disrupts tissue integrity. OTM involves alveolar bone and periodontal tissues. The administered force compresses one side of the PDL and stretches the other.^40^ The orthodontic force during OTM leads to signals that promote endothelial cell migration to leukocytes, which release signaling molecules such as cytokines, chemokines, growth factors, and inflammatory mediators to remodel the ECM in PDL and alveolar bone. The results obtained in the present study demonstrated that the administration of banana leaf extract nanoemulsion significantly enhanced the expression levels of bone formation markers comprising ALP, osteocalcin, VEGF, and CD31 on the tension side during OTM. This effect was consistently superior to the orthodontic force only treatment and comparable to or higher than that of the reference drug etoricoxib at several observation times. These findings suggest that the banana leaf extract had a biologically meaningful osteogenic effect during OTM.

Banana leaves contain a complex phytochemical matrix that is rich in flavonoids (quercetin and catechin), tannins, saponins, terpenoids, and alkaloids.^24^, ^25^, ^26^ These compounds do not work in isolation, but instead their synergistic interactions may create an environment that is highly favorable for osteoblast activation and bone remodeling.^23^ The simultaneous increase in ALP, an initial osteoblastic differentiation marker, and osteocalcin, a sign of late-stage bone maturation, demonstrate that the extract modulated bone remodeling in a coordinated and time-dependent manner.^20^, ^21^, ^22^^,^^34^

In the present study, the banana leaf extract increased the osteoblastic activity during OTM according to the significant upregulation of ALP in the initial phase and osteocalcin in the next phase. These changes show that the phytochemical constituents in the extract helped to amplify osteoblast differentiation and maturation more than the effects induced by mechanical loading alone.^23^^,^^27^^,^^28^ The mechanical tension produced by orthodontic force triggers a cascade of responses in the PDL.^8^, ^9^, ^10^ Stretching induces PDL activation and upregulates the expression of COX-2, PGE_2_, and VEGF.^18^^,^^29^, ^30^, ^31^, ^32^, ^33^ These changes increase local angiogenesis and recruit mesenchymal stem cells to the tensile area, providing space for their differentiation into target cells in the osteoblast lineage.^17^, ^18^, ^19^, ^20^ In the present study, this physiological response appeared to be enhanced by the bioactive compounds in the banana leaf extract.^23^^,^^27^^,^^28^

Flavonoids and other polyphenolic compounds in banana leaf extract have been shown to possess antioxidant and anti-inflammatory properties that could support tissue repair and bone remodeling.^23^ These bioactive compounds may create a favorable biological environment for angiogenesis and osteoblast function. In the present study, the significantly increased expression levels of VEGF and CD31 indicated enhanced angiogenic activity, and the increased ALP and osteocalcin expression levels suggested enhanced osteoblast differentiation and bone formation.^23^^,^^28^ The underlying molecular pathways were not investigated in this study but the coordinated increases in angiogenic and osteogenic markers suggest that the banana leaf extract nanoemulsion may have promoted periodontal tissue remodeling during OTM.

The combined antioxidant, anti-inflammatory, and pro-osteogenic effects of banana leaf phytochemicals produced a biochemical environment that was favorable for osteoblast differentiation and activity.^23^ This synergistic mechanism led to the observed increases in both initial-stage markers (ALP and osteocalcin), showing that the banana lead extract accelerated initial osteoblast activation and also maintained mineralization over time.^20^, ^21^, ^22^ These findings suggest that banana leaf extract can potentially be applied as an adjunctive therapeutic agent to maximize bone remodeling during orthodontic treatment.^36^^,^^37^

Tannins in banana leaves contribute to enhancing collagen matrix production, stabilizing extracellular proteins, and promoting mineral deposition by interacting with calcium ions.^23^, ^24^, ^25^ In addition, saponins and tannins have been reported to contribute to tissue repair by promoting extracellular matrix organization and supporting angiogenesis. Enhanced vascularization facilitates oxygen and nutrient delivery to the PDL, creating conditions that favor osteoblast activity and new bone formation. Similarly, in the present study, the banana leaf extract resulted in significantly higher VEGF and CD31 expression levels, which were accompanied by increased ALP and osteocalcin expression levels throughout the observation period.^27^ These combined effects explain the great increases in osteocalcin expression under KP, particularly those observed on days 21–28. Terpenoids may enhance VEGF expression and microvascular development.^26^ During OTM, angiogenesis is critical for the nutrient supply, osteoprogenitor recruitment, and osteoblast differentiation on the tension side.^18^, ^19^, ^20^ Enhanced vascularization synergizes with osteogenic activity, generating signals that accelerate bone formation, as shown by the increased ALP and osteocalcin levels in the present study. The mechanical tension applied to the tooth elicits a mechanotransduction response in the PDL and this force activates fibroblasts in the PDL, causing COX-2, PGE_2_, and VEGF upregulation, which collectively drive the initial inflammatory and angiogenic signaling needed for OTM.^29^, ^30^, ^31^, ^32^, ^33^ PGE_2_ and VEGF then facilitate the recruitment of mesenchymal stem cells and their migration to the tension area, where they differentiate into osteoblast lineage cells.^17^ The biological effects observed in the present study were probably related to the combined effect of the bioactive phytochemicals present in the banana leaf extract. These compounds may support angiogenesis and periodontal tissue remodeling by enhancing the endothelial activity and maintaining a favorable microenvironment for osteoblast differentiation. This interpretation is supported by the concurrent upregulation of VEGF and CD31 as angiogenic markers, as well as the increases in ALP and osteocalcin expression, which are indicators of osteoblast activity and bone formation.^36^^,^^37^

The initial osteoblastic response characterized by enhanced ALP expression demonstrated maturation of the active matrix followed by late enhancement, thereby signaling progression to extracellular matrix mineralization and the completion of new bone formation on the tension side.^20^, ^21^, ^22^ Thus, the extract may function as an anti-inflammatory modulator and potentially as a bone anabolic enhancer. This multi-targeted mechanism differentiates it from etoricoxib, which primarily targets inflammatory pathways but may also suppress long-term osteoblastic activity.^33^^,^^35^

In the present study, the VEGF and CD31 expression levels on the tensile side were higher under treatment with banana leaf extract compared with the control. Bone vascularization is modulated by VEGF. Tannin in the banana leaf extract may have increased angiogenesis, promoting the production of fibroblasts and facilitating the deposition of collagen, as well as wound contraction.^41^ Angiogenesis is important in bone remodeling and formation via osteogenesis and it is organized by angiogenic and osteogenic genes, including VEGF, fibroblast growth factors, and bone morphogenic proteins.^38^

Saponins can stimulate VEGF and increase the migration of macrophages to the wound site, leading to the production of cytokines that activate fibroblasts in the wound tissue. Saponins can play roles as angiogenic agents by modulating VEGF to increase the mitogenic activity of endothelial cells in the vascular development proliferative phase. VEGF can also function to increase angiogenesis during wound curing.^41^ The expression of VEGF was recorded throughout osteoblast differentiation, and VEGF is a critical cytokine released by the bone matrix during the bone remodeling process. The increased expression of VEGF may enhance the transport of nutrients and oxygen via blood vessels.^39^ Throughout OTM, compressive forces promote angiogenesis in the PDL mediated by VEGF. VEGF is important in PDL remodeling and also implicated in bone resorption and creation.^42^

On day 14, VEGF expression was enhanced, probably due to the increases in osteoclasts in the region throughout the first days of OTM, which can be explained by VEGF promoting the differentiation of osteoclast. Ongoing compressive forces increased VEGF synthesis and the angiogenic activity in PDL cells, potentially facilitating periodontal remodeling during OTM. Studies indicate that VEGF expression in compressed periodontal tissue may significantly influence bone resorption and facilitate angiogenesis in hyalinized tissues and neighboring regions on the pressure side. Furthermore, the effects of VEGF on vascular permeability and chemotaxis may help to supply degraded tissues with various cell types, such as fibroblasts, macrophages, and multinucleated giant cells.^43^

Platelet/endothelial cell adhesion molecule-1 (PECAM-1) was first identified in the mid-1980s as the CD31 differentiation antigen present on the surface of human granulocytes, monocytes, and platelets.^44^ PECAM-1 mediates interactions between leukocytes and endothelial cells, localizing at endothelial cell junctions, where it serves as a primary mechanosensor and regulator of vascular permeability. CD31 is predominantly expressed in vascular endothelial cells. VEGF regulates vascular neogenesis, which then facilitates bone neogenesis, indicating that VEGF is a crucial factor in the bone formation process. CD31 serves as a marker for vascular endothelial cells, facilitating the inference of angiogenesis by analyzing VEGF and CD31 expression levels.^45^

Etoricoxib, a selective COX-2 inhibitor, was used as a pharmacological reference in the present study due to its ability to modulate inflammation without severely impairing bone remodeling at therapeutic doses.^29^, ^30^, ^31^ In this study, etoricoxib moderately increased the ALP and osteocalcin levels, but the banana leaf extract promoted greater osteogenic enhancement, especially on days 21–28. The superior performance of the extract was probably due to its multi-targeted biomolecular synergy rather than single-pathway inhibition.^27^^,^^28^^,^^36^ In contrast to etoricoxib, which modulates prostaglandin synthesis, banana leaf extract simultaneously influences oxidative stress, inflammatory signaling, osteogenic transcription, and angiogenesis.

A limitation of the present study was the lack of a vehicle control group receiving a blank nanoemulsion formulation without banana leaf extract. Therefore, the potential influence of the nanoemulsion carrier system itself on the local tissue response, angiogenesis, and bone remodeling cannot be completely excluded. Future studies should include a vehicle control group to distinguish the biological effects of the active extract from those of the nanoemulsion delivery system. In addition, pairwise comparisons were evaluated using Fisher's LSD post hoc test after one-way ANOVA detected statistically significant differences, and this is a common approach applied in exploratory experimental studies. Future studies with larger sample sizes may also consider employing complementary conservative post hoc procedures, such as Tukey's test or Bonferroni's adjustment, to further reinforce the statistical confidence when evaluating multiple comparisons.

In this study, activity during OTM was demonstrated by marked increases in the ALP, osteocalcin, VEGF, and CD31 expression levels. The synergy among phytochemicals in the extract, particularly flavonoids, may have supported osteogenesis through antioxidant, anti-inflammatory, and pro-osteogenic molecular pathways that complemented mechanical tension-induced remodeling. Compared with mechanical force alone and the reference drug etoricoxib, the banana leaf extract produced superior early (ALP) and late (osteocalcin) osteoblastic responses. These findings suggest that banana leaf extract nanoemulsion may be applied as a promising adjunct to enhance bone remodeling biomarkers associated with OTM.

## Ethical approval

The animal experimental protocol in this study was approved by the Ethics Committee for Animal Experiments of the Faculty of Dental Medicine, Universitas Airlangga (Approval No. 0401/HRECC.FODM/III/2025). The study was conducted in accordance with the ARRIVE guidelines and internationally accepted principles for animal care and use.

## Authors contributions

AT: Conceptualization, funding acquisition, data curation, investigation, methodology, writing - original draft, review, and editing. NARP: Conceptualization, project administration, supervision, validation. RYR and NZS: Investigation, methodology, data curation, writing - original draft. IGP: investigation, writing - original draft. IGAWA and ERW: Supervision, validation. WR: Investigation, resources. DN, LFZ, AFP, and TNE: Visualization. AP: Funding acquisition. APN: Conceptualization, methodology, supervision, validation, writing - review & editing. All authors have critically reviewed and approved the final draft and are responsible for the content and similarity index of the manuscript.

## Source of funding

The authors acknowledge support from the Airlangga Research Fund, Faculty of Dental Medicine at Universitas Airlangga with contract number 1798.UN3.LPPM/PT.01.03/2025 and the Decree of the Rector Number 372/UN3/2025.

## Conflict of interest

The author(s) have no conflict of interest to declare.
